# Supplementary material for: Are carbon emissions trading and green financial instruments synergistic? -Comprehensive quantitative research based on content analysis
Source: PLoS One. 2024 Mar 7;19(3):e0298601. doi: 10.1371/journal.pone.0298601 (PMC10919601; doi:10.1371/journal.pone.0298601)
Supplement: S1 Data — (ZIP) [file pone.0298601.s001.zip › Data/地级市Z_F工作报告.zip!/╣π╬≈/▒▒║ú╩╨/└┤╫╘╣1⁄2╓┌║┼í╛┬φ┐╦╩2╛▌═°í┐_2017.docx]

各位代表：

现在，我代表北海市人民政府，向大会报告政府工作，请予审议，并请市政协委员和列席会议的同志提出意见。

一、2016年工作回顾

过去一年，面对严峻复杂的宏观经济形势和持续加大的经济下行压力，在自治区党委、政府和市委的正确领导下，我们紧紧围绕市十四届人大七次会议确定的目标任务，主动适应经济发展新常态，积极应对困难与挑战，凝心聚力、团结奋进，保持了经济社会较快健康发展，实现了“十三五”发展良好开局。

主要工作和成效体现在以下十二个方面：

（一）综合实力获得新提升。坚持以发展经济为主线，积极落实稳增长各项政策，多项经济指标排全区前列，全市经济发展实现“四个突破”。地区生产总值突破千亿元，达到1007.3亿元、增长8.6%；规模以上工业总产值突破两千亿元，达到2180.7亿元、增长16.4%，规模以上工业增加值完成448.6亿元、增长10.3%；固定资产投资突破千亿元，达到1011.1亿元、增长9.9%；财政收入完成166.3亿元、增长16.3%，国税收入突破百亿元，成为全区第四个国税收入超过100亿元的城市；外贸进出口总额205亿元，社会消费品零售总额225亿元、增长11%，城镇居民人均可支配收入29412元、增长6.9%，农村居民人均可支配收入11622元、增长9.4%，城镇新增就业2.19万人，城镇登记失业率3.15%、低于年度控制数0.85个百分点。

（二）主导产业实现新发展。电子信息、石油化工、临港新材料三大产业继续保持快速发展势头，合计完成工业产值1866.3亿元、增长18%，占全市规模以上工业总产值的85.6%。电子信息制造业完成产值1174.5亿元、增长18.4%；石油化工产业完成产值303.1亿元、增长17.5%，原油加工能力从500万吨提升至650万吨；临港新材料产业完成产值388.7亿元、增长16.9%。大力推进项目建设，全年实施重点项目266个，新开工项目70个，林纸一体化一期工程和年产20万吨热磨机械木浆生产线、中石化广西LNG一期、诚德不锈钢冷轧、星沅LCM等一批项目竣工投产，新的经济增长点加快形成。

（三）园区经济集聚新动力。园区的产业承载能力不断增强，集聚效应进一步凸显，合计完成产值2052.2亿元、增长17.7%，占全市规模以上工业总产值的94%。北海工业园区完成产值750.1亿元、增长19.2%，其中电子信息制造业产值占全市的61.2%，成为电子信息产业发展的主力。铁山港（临海）工业区完成产值725.5亿元、增长16.8%，石油化工、临港新材料两大产业挑起临海工业的“大梁”。北海出口加工区完成产值373.1亿元、增长16.4%，加工贸易进出口54.6亿元，成为北海发展外向型经济的“拳头”。北海高新区完成产值100.8亿元、增长16.9%，聚集了一批科技型企业。合浦工业园完成产值102.7亿元、增长17.1%，北海海洋产业科技园获评“国家科技兴海产业示范基地”，铁山东港产业园规划建设加快推进。

（四）城市建设管理出现新变化。完成城市总体规划修编和综合交通、市政、蓝线、绿线、防洪排涝、海岸线功能景观专项规划编制工作。上海路跨铁路立交桥、重庆路（广东路至湖南路段）、金海岸大道改造等一批路桥项目竣工通车，“七纵七横”的城市主干路网基本贯通。铁路排水明渠西段建成，北海大道排水改造工程初步完成，主城区内涝问题基本解决。实施城市环境综合整治百日行动，市容市貌和道路交通秩序明显改善，得到市民和游客的好评。

主动认领、照单全收中央环保督察组反馈的督察意见，严肃查处和整治了一批环境突出问题，扎实推进合浦采石场破坏生态环境问题整改工作。环境质量保持良好，空气质量优良率达95.6%，城市集中式饮用水水源地水质达标率100%，海水浴场水质优良率100%，海洋生态系统处于健康状态，北海滨海国家湿地公园成为广西首家国家湿地公园。

（五）旅游及服务业发展进入新阶段。北海入选首批国家全域旅游示范区创建城市。全年接待国内旅游者2473万人次、增长15.4%，接待入境旅游者13.6万人次、增长5%，国内旅游消费284亿元、增长29.4%，国际旅游消费5571万美元、增长9.5%。银滩旅游度假区管理体制调整完成，景区综合整治初显成效。涠洲岛入选中国“十大美丽海岛”，火山博物馆新馆主体建筑等项目基本竣工。金海湾红树林景区获评“全国生态文化示范基地”，合浦汉闾文化园获评国家4A级景区。成功创建3家四星级乡村旅游区和两家五星级农家乐。曲樟璋嘉客家老屋村、营盘白龙村入选全国第四批传统村落。

商贸物流等服务业加快发展，多个大型商贸综合体项目顺利推进，电商集聚区建设加快，新增限额以上商贸统计企业117家，在北部湾股交所新增挂牌企业63家，全市港口货物吞吐量完成2750万吨、增长11.4%。新建商品房销售面积222.6万平方米、增长13.7%，库存商品住宅消化周期15.4个月，房地产“去库存”取得明显成效。

（六）脱贫攻坚赢得新战果。全市有22个贫困村达标实现脱贫摘帽，有15569人通过“双认定”达到脱贫标准，完成自治区下达的年度脱贫6574人的任务。全市整合资金共8.89亿元用于脱贫攻坚，贫困村基础设施进一步完善，产业扶贫覆盖全部贫困户，1370户贫困户完成危房改造，2810户贫困户获得扶贫小额贷款，农村低保对脱贫攻坚的贡献率从12%提高到42.1%，“百企扶贫”行动扎实开展，脱贫攻坚结对帮扶实现全覆盖。

（七）农村农业呈现新气象。农林牧渔业增加值增长4.2%。成功创建自治区和市、县（区）、乡（镇）四级现代农业核心示范区16个。水产品总产量110.6万吨，罗非鱼、对虾等示范养殖面积达24万亩。水利固定资产投资完成13.8亿元，建成标准海河堤11.7公里，解决农村5.1万人的饮水安全问题，完成36个水库移民新村建设。“美丽北海·生态乡村”建设投入资金1.57亿元，完成1953个村屯绿化，农村人居环境进一步改善。

（八）改革创新取得新进展。扎实推进供给侧结构性改革，行政审批项目从287项减少到256项，全部取消非行政许可审批事项，降低企业成本约9亿元。在全区率先推进“六证合一、一照一码”，企业证照办结时间从20个工作日缩减至5个工作日。提前完成自治区下达的国库支付电子化试点改革任务。全面推开“营改增”改革试点，为纳税人减负约6亿元。新增国家级高新技术企业10家，中电北海产业园众创空间、高新区21度聚变空间被认定为国家级众创空间。

（九）开放合作注入新活力。新引进项目194个，新签项目合同投资额1078亿元，实际到位资金388.2亿元（含续建项目），加工贸易进出口总额119亿元，全口径实际利用外资4.98亿美元、增长15.4%。北海机场已开通20条航线，通达25个城市，全年旅客吞吐量123万人次，创历史新高。进出北海动车车次最多时每天达27对，全年客运到发量突破700万人次。北海港口岸扩大开放通过自治区预验收，合湛铁路、松旺至铁山港东岸高速公路开工建设，铁山港北暮作业区5#-6#泊位竣工。成功举办2016年世界海洋日暨全国海洋宣传日主场活动、汉代海上丝绸之路考古与汉文化国际学术研讨会。

（十）民生改善增添新成效。一般公共预算用于民生领域的支出超过75%。16件为民办实事事项基本落实，改革发展的成果更多惠及全市人民。新建、改扩建中小学和幼儿园163所，铁山港区义务教育均衡发展通过自治区督导评估。深入实施“文化惠民”工程，新建村级公共服务中心29个，文化惠民演出180场，农村公益电影放映4116场。碧海丝路大剧院投入使用，工人文化宫改扩建、图书馆新馆、体育馆等项目开工建设。传染病防控工作进一步加强，首批通过自治区消除疟疾考核评估；基层医疗卫生服务能力建设得到加强，派出市级医疗队驻涠洲岛为群众提供医疗服务；我国首支国家级海（水）上紧急医学救援队伍落户北海；鼓励社会力量办医，新增妇产科病床120张。

社会保障持续完善，城市低保标准从每人每年5040元提高到5760元，农村低保标准从每人每年2600元提高到3000元。社会保险待遇水平持续提高，居民医保政府补助从380元提高到420元，职工医保年度最高报销限额提高9.9%，实现区内异地就医结算。全市各项社会保险参保人数达到164.8万人，基本实现全覆盖。调增企业退休人员基本养老金，增幅7.6%。保障性住房和棚户区住房改造新开工3958套（户）、基本建成4879套、分配入住2037套。外事、侨务、民族、宗教、新闻出版、档案、地方志、妇女儿童、残疾人等方面工作进一步加强。

（十一）社会治理得到新加强。推进全面依法治市，深入开展法治宣传教育，经济社会建设法治化水平进一步提高。平安北海建设扎实推进，主要治安指数实现“七升七降”，社会治安持续好转。2016年，群众安全感单项排名提升到全区第八、综合排名第六，群众对公安队伍的满意度全区排名第三，创近年来最好成绩。基层应急能力不断增强，突发事件得到有效处置。开展食品药品专项整治，规范食品药品生产经营秩序，保障人民群众饮食用药的安全。严格落实安全生产责任，加强排查整治，安全生产形势平稳。国防动员、人民防空和后备力量建设、双拥共建工作扎实推进。

（十二）政府建设展现新作为。全面落实从严治党主体责任，深入开展“两学一做”学习教育，坚持不懈抓好作风建设，政府的行政效能和服务水平进一步提升。深入推进党风廉政建设，强化行政监察和审计监督，加强扶贫领域监督执纪问责。在全区率先实现政府权力清单和责任清单市、县（区）、乡（镇）三级全覆盖，全市45个部门的权力事项由5929项压缩到3436项，精简率达42%。自觉接受市人大、市政协和社会监督，人大代表建议、政协提案办结率和满意率均达100%。

各位代表！

过去一年，我们化解压力、团结拼搏、砥砺奋进，成绩来之不易、进步难能可贵。这是自治区党委、政府和市委统揽全局、正确领导的结果，是市人大及其常委会和市政协有效监督、大力支持的结果，是全市人民齐心协力、真抓实干的结果。在此，我谨代表市人民政府，向全市广大干部群众，向各民主党派、各人民团体、各界人士和驻市单位、驻市部队、武警官兵，向所有关心、支持北海建设发展的同志们、朋友们，表示衷心感谢，致以崇高敬意！

我们也清醒地认识到，北海的发展仍然存在不少困难和问题。重大项目储备不足，一些项目建设进展不快，固定资产投资增速放缓，新增长点不多、新动力不足；城市管理水平不高，市容环境亟待改善；开放平台建设滞后，进出口增幅放缓，对外开放总体水平不高；央视曝光的合浦采石场破坏生态环境等问题比较突出，生态环境保护基础性工作存在不少问题；政府系统少数工作人员思想解放不够，面对经济新常态推动发展的办法不多、能力不强；一些干部担当意识不强，仍存在不作为、不能为、不善为等现象；一些领域仍存在审批把关不严、事中事后监管不力等问题。

面对这些问题，我们不回避、不推诿、不遮掩，将采取积极有效措施，切实加以解决。

二、2017年工作总体要求和主要目标

今年1月15日，自治区党委书记、自治区人大常委会主任彭清华同志参加自治区十二届人大六次会议北海代表团分组审议，在肯定北海工作的同时，对北海今后发展提出了殷切期望，要求北海进一步找准定位、发挥优势，舞起北部湾经济区扩大开放合作的龙头、临港产业尤其是电子信息产业升级发展的龙头、海洋经济发展的龙头和滨海旅游发展的龙头，当好广西排头兵。这是自治区党委、政府对北海发展的新定位、新要求，符合北海发展的实际，符合人民群众的期待，为北海今后的发展指明了方向、提供了遵循。

我们一定要落实自治区党委、政府对北海发展的新要求，增强舞起“四个龙头”的责任感、使命感和紧迫感，振奋精神、凝心聚力，按照市委十一届三次全会确定的目标任务，全力以赴做好政府各项工作，为广西营造“三大生态”、实现“两个建成”做出更大贡献。

今年政府工作的总体要求是：全面贯彻党的十八大和十八届三中、四中、五中、六中全会以及中央经济工作会议精神，深入学习贯彻习近平总书记系列重要讲话精神和治国理政新理念新思想新战略，落实自治区党委、政府和市委的决策部署，坚持稳中求进工作总基调，牢固树立和贯彻落实新发展理念，扎实推进供给侧结构性改革，加快发展实体经济，深入实施“四大会战”，奋力舞起“四个龙头”，促进经济持续较快健康发展和社会和谐稳定，加快建设创新型开放城市，以优异成绩迎接党的十九大胜利召开。

今年全市经济社会发展的主要预期目标是：地区生产总值增长8.6%以上，财政收入增长10%，规模以上工业增加值增长11%，固定资产投资增长10%，外贸进出口总额增长10%，社会消费品零售总额增长10%，城镇居民人均可支配收入增长9%，农村居民人均可支配收入增长10%，城镇新增就业2万人，城镇登记失业率控制在4%以下，居民消费价格指数涨幅控制在3%左右，完成节能减排任务。

上述主要经济指标增速总体上高于全区水平，符合市委十一届三次全会的部署要求。实现上述目标，既不可能一蹴而就，也不是高不可攀。我们必须增强信心、铆足劲头、攻坚克难，撸起袖子加油干，才能确保完成任务。

面对新形势、新任务、新要求，要继续保持北海经济社会较快健康发展，完成全年目标任务，我们必须把握好“八个更加重视”：

一是更加重视发展第一要务，抓住发展壮大产业不放松。发展是硬道理硬任务，加快发展仍然是解决北海所有问题的关键。北海的当务之急，就是迅速“做大蛋糕”，解决总量小、财力薄、产业结构抗风险能力弱的突出问题。我们必须以经济发展为主线，紧紧抓住产业这个“牛鼻子”，大力发展实体经济，壮大优势产业，扩大经济规模，提升发展质量和效益，筑牢城市发展的产业支撑，不断增强北海的综合实力和竞争力。

二是更加重视继续解放思想，抓住创新驱动发展不放松。创新是引领发展的第一动力。我们必须继续解放思想，打破思想禁锢，更新思想观念，努力营造掀起新一轮发展的舆论氛围，用创新思维破除制约发展的体制、机制瓶颈。要依靠创新驱动，善于借鉴、学习先进地区的做法和经验，引进和培养创新型人才，落实促进创新创业政策措施，大力发展新技术、新业态、新模式、新产业，培育新的经济增长点，开辟发展的新空间，增强发展的新动力。

三是更加重视增强发展后劲，抓住扩大项目投资不放松。项目是硬载体硬支撑，是产业发展的根本。项目投资是增强发展后劲的源头，是北海现阶段拉动经济增长最直接、最有力、最有效的第一驾马车。我们必须强化“抓项目投资就是抓发展”的意识，轰轰烈烈抓招商，扎扎实实推项目，培育优势产业，扩大有效投资，促进企业发展，加快形成“大企业顶天立地，小企业铺天盖地”的格局，夯实城市发展的后劲，实现有质量的可持续发展。

四是更加重视深化开放合作，抓住融入北部湾发展不放松。开放是北海最大的优势，是北海加快发展的必由之路。我们必须站在全国全区的高度谋划北海的开放发展，积极参与“一带一路”建设，深化与东盟和粤港澳台的合作，深度融入北部湾城市群建设和北部湾经济区开放开发，舞起北部湾经济区扩大开放合作的龙头。以更大的投入推进公路、铁路、港口码头建设，以更多的方式创新和建设开放合作平台，以更强的力度发展外向型经济，实现更高水平的开放发展。

五是更加重视生态文明建设，抓住整治环境问题不放松。坚持绿色发展，顺应发展的新要求和群众的新期待，牢固树立“绿水青山就是金山银山”的强烈意识，守住生态环境的底线。我们必须正确处理好经济发展同生态环境保护的关系,加大自然生态系统和生态环境保护力度，重点整治群众反映强烈的环境突出问题，巩固发展北海生态环境的优势和竞争力，建设海碧天蓝、山清水秀的美丽生态家园。

六是更加重视统筹城乡发展，抓住壮大县域经济不放松。坚持协调发展，积极推动城乡一体化发展，努力缩小城乡差别、补齐发展短板。我们必须把壮大县域经济作为统筹城乡发展的重要抓手，增强县区发展能力，提升县区经济实力。加大支持农村发展的投入力度，推动城市基础设施和公共服务向县区、向农村延伸。把合浦的发展纳入全市发展大局，统筹布局、统筹推进、协调发展。加大对市辖区发展的政策支持力度，解决市辖区自主权不足、自我积累能力不强等问题，促进市辖区加快发展。

七是更加重视增进民生福祉，抓住保障改善民生不放松。坚持共享发展，继续加大对民生领域的投入，增强公共服务能力，推进基本公共服务均等化。我们必须着力解决事关群众切身利益的具体问题，扎实推进精准脱贫，繁荣发展社会事业，健全完善保障体系，创新社会治理，建设平安北海，维护社会和谐稳定，不断提高群众生活水平，让全市人民共享更多的改革发展成果。

八是更加重视政府自身建设，抓住改进工作作风不放松。打铁还需自身硬。面对新形势、新任务，加强政府自身建设、转变干部作风显得尤为重要。我们必须进一步转变政府职能，坚持依法行政，深化简政放权，优化政务服务，提高办事效率，切实提升政府服务能力和水平。要持之以恒加强作风建设，大力弘扬“我不上谁上”的担当精神，真抓实干、开拓进取、奋勇争先，把中央的政策和市委的部署贯彻到位，把各项工作落到实处，创造新业绩，推动新发展。

三、2017年工作安排

新的一年，既要统筹抓好稳增长、促改革、调结构、防风险、惠民生各项工作，又要突出重点，针对发展所需、群众所盼的事项，着力抓好落实、务必抓出成效。我们将重点做好十五个方面的工作：

第一，加快工业转型升级发展。落实供给侧结构性改革要求，着力发展实体经济，全面实施工业转型升级大会战，以质量效益为基础，优化存量、扩大增量，完善产业配套，坚定不移做大做强主导产业，推动传统优势产业转型升级，奋力舞起临港产业尤其是电子信息产业升级发展的龙头。

推动电子信息产业创新升级发展，电子信息制造业实现产值1350亿元。实施电子信息产业发展倍增计划，重点推进三诺、惠科、硕华、京凯达等项目建设，加快建设中国东盟信息港北海基地、中电北部湾信息港、高新区软件和信息服务业基地、北部湾表面处理中心等平台项目，升级建设电子信息产品检验检测中心，推动电子信息产业由中低端向中高端发展，促进电子信息制造业向智能化转型、向软件开发和研发转型，加快打造全国重要的电子信息产业基地。

推动石油化工产业延伸发展，实现产值350亿元。重点推进中石化北海炼化质量升级项目，积极推进广西LNG项目二期、新鑫能源、中航化等一批项目建设，引进一批新的石化配套项目，延伸石化产业链、扩大产业集群。

推动临港新材料产业增量发展，实现产值450亿元。重点发展不锈钢冷轧薄板及制品，支持企业拓展潜在的不锈钢消费市场，扩大规模。积极研究推进战略性储能新材料产业，推动中储能项目开展前期工作，争取规划建设储能新材料产业园。

推动林纸和食品产业提升发展。重点推进北海林纸一体化一期工程和年产20万吨热磨机械木浆生产线尽快达产，加快发展成为主导产业之一。着力发展水产品精深加工业，引导水产品企业升级改造，提升产业层次。依托临港优势，积极发展粮油加工业，提高生产能力，扩大产业规模。加快月饼小镇等项目建设，推动农产品加工业向精深加工集聚发展，壮大合浦食品产业。

推动新兴产业加快发展。积极培育发展新一代信息技术、先进装备制造、新材料、新能源、生物医药、节能环保等产业，扶持和引进新兴骨干企业，打造新的拳头产品，进一步提升新兴产业在工业中的比重，推动新兴产业成为工业发展新动能。

推动产城融合发展。坚持区城共建、产城融合，推动园区和新城区融合发展，把产业园区建设成为产业集聚、功能完善、环境宜居的产业新城。科学规划新城区和产业园区功能布局，统筹生活区、商务区、办公区等城市功能建设，完善园区基础设施体系，加快建设园区的医院、学校、农贸市场、综合商场等配套设施，形成以产兴城、依城促产、产城互动发展格局。

推动电力基础设施建设。建成500千伏北海变电站，加快改造提升北海电网基础设施，提高电网供电能力和供电可靠性，满足北海建设发展的电力需要。

第二，加强城乡规划建设管理。实施城乡建设管理大会战，开展“城乡环境提升年”活动，实施“十大提升行动”，巩固城市环境综合整治百日行动成果，进一步遏制城市环境乱象回潮反弹，加快建设美丽宜居北海。

实施城市规划提升行动。进一步完善城市规划体系，凸显滨海特色的城市建筑风格，完成商业网点（包括农贸和专业市场）、消防、学校、综合管廊、绿道、路名等专项规划，启动城市照明、户外广告、停车场等专项规划。开展片区控制性详细规划编制和城市设计。

实施交通秩序提升行动。科学施划停车泊位，整治与合理设置车辆停放点、停车场，规范车辆停放秩序。优化交通组织，统筹安排执法勤务，提高道路见警率，继续对“四车”违法和车辆、行人交通违法行为进行整治，实现城市道路交通秩序根本好转。

实施市容环境提升行动。落实市容环卫“门前三包”责任制，完善环卫保洁工作机制，力争道路机械化清扫率从49%提高到80%。开工建设生活垃圾焚烧发电、餐厨垃圾处理项目，新建、改建一批公共厕所。规范户外广告审批和设置，开展北京路户外广告设置示范试点。综合治理城市扬尘和废气、油烟污染。

实施市政园林提升行动。新建和改造20条、续建8条市政道路，改造提升30条小街小巷，开工建设冯家江和西村港大桥。提标改造红坎、合浦和铁山港污水处理厂，推进市区56个雨污合流排放口截流改造。推进城市地下综合管廊和涠洲岛、海洋产业科技园区等区域的海绵城市示范项目。在全区率先建成市区WiFi全覆盖和免费WiFi上网的城市。启动“黄金北岸”项目，加快市区北岸景观再造和旅游步道建设；推进“北海绿廊”建设，沿市区铁路线规划建设绿色长廊、休闲步道和带状公园。

实施土地盘活利用提升行动。根据城市规划和经济发展需要，积极盘活市辖区低效利用和闲置的工业、仓储用地，鼓励将工业、仓储厂房用于产业转型升级，解决职工安置问题。按照规划要求和村民意愿，通过收储、委托出让、合作开发等方式，加快盘活和开发利用集体就业用地，确保被征地农民长远生计有保障和集体经济组织有稳定收益。加快推进“城中村”改造，对集中成片棚户区、集中连片城镇旧住宅区、非成片棚户区等进行分类、分步改造，改善群众居住条件。

实施市场建设提升行动。用三年时间开展一场市场革命。规划新建一批市场，加快建设蔬菜和水果批发市场、建材市场等专业市场，推进大型商业综合体尽快建成营运。改造提升一批市场，加强市场标准化、规范化建设，改造升级市场摊位、消防、通风、排水和监控等设施。整治规范一批市场，对市场周边的违法建筑、乱摆卖等乱象进行治理，营造整洁卫生、规范有序、公平交易的经营环境。

实施小区管理提升行动。规范物业管理企业行为，鼓励推广小区业主自治管理模式。强化物业专项维修资金使用的监督管理，整治侵犯业主合法权益、破坏公共绿地、侵占公共区域等行为。开展“和谐小区”评选，以点带面提升小区管理水平。

实施村镇建设提升行动。推进南康镇“中国特色小镇”建设，实施公馆、福成、西场三镇的广西百镇建设示范工程，加强保护和修复传统村落、特色名村。深入开展“美丽北海·宜居乡村”活动，重点实施“产业富民”“基础便民”“服务惠民”专项活动，完善村镇保洁机制，推进农村垃圾专项治理两年攻坚行动，完成农村改厨改厕试点任务。

实施城市管理能力提升行动。统筹推进城市的规划、建设、管理，发挥各职能部门的作用，激发公众参与，形成城市综合管理的合力。整合现有信息化资源，建成数字化城市管理平台，推进城市网格化和精细化管理。加强城市综合管理队伍建设，推行城市综合执法，提高文明执法能力和水平。

实施市民文明素质提升行动。深入开展文明城市创建活动，培育和践行社会主义核心价值观，出台市民“文明公约”，促进环境卫生督查、文明劝导常态化，引导和规范市民文明行为。树立先进模范典型，鼓励志愿者公益行动，引领文明风尚，全面提升市民文明素质和城市文明程度。

第三，推动旅游建设发展。全面实施全域旅游城市建设大会战，奋力舞起滨海旅游发展的龙头，加快打造区域性国际化滨海旅游名城。力争接待国内旅游者2700万人次、增长10%，实现国内旅游消费340亿元、增长20%。

加快推进银滩和涠洲岛开发建设。北海银滩是广西旅游的金字招牌，更是北海旅游的金字招牌，要以创5A为目标，以国际视野、崭新理念和生态化目标规划设计，大手笔、大动作推进银滩中区建设。加快推进搬迁工作，把银滩北背岭回建区打造成为富有渔家风情特色的“疍家小镇”，引导搬迁群众转产转业，加快发展特色产业。持续推进银滩区域环境综合整治，完善严管严控长效机制，开展银滩中区生态修复，提档升级银滩景区基础设施，确保银滩区域环境质量、公共秩序明显改善，推动银滩景区提高品质、丰富内涵。以民俗风情、生态旅游为方向加快涠洲岛开发建设，开展涠洲岛概念规划方案国际竞标和总体规划修编，加快建设道路、供水等基础设施，推进涠洲岛高端度假酒店和旅游设施项目建设，依法控制和清理违章建筑，进一步规范旅游市场秩序。

加快推进旅游景区和设施建设。编制完成《北海全域旅游总体规划》。大力推进涠洲岛鳄鱼山、北海银滩、园博园、金海湾红树林景区创建国家5A级旅游景区工作，加快建设合浦汉文化国家考古遗址公园、北海工艺美术博览园，规划建设廉州湾滨海旅游带。开工建设一批高等级度假酒店和特色民宿酒店，启动建设三个主题乐园。规划建设北部湾旅游集散中心，推进旅游厕所改造和旅游标识系统建设。支持合浦县创建广西特色旅游名县，加快侨港、高德、曲樟等特色旅游小镇建设。

加快开发旅游产品和塑造品牌形象。依托北海旅游资源，加快开发海岛体验、滨海休闲、海洋运动等滨海休闲度假旅游产品，促进文化体育与旅游产业融合，大力培育发展乡村旅游，丰富旅游产品结构。利用媒体平台、旅游会展，加强旅游形象宣传和市场营销，扩大北海旅游品牌影响。动员多方力量，举办主题旅游文化节庆和旅游体育赛事。

加快推进旅游开放合作。积极策划打造面向东盟的旅游开放合作平台。加快建设北部湾国际邮轮母港，推进北海港石步岭港区货运功能向铁山港区转移，推动中越海上旅游航线复航。培育发展航空航线，加密重点客源城市和地区的旅游航班。

大力整治旅游市场秩序。健全旅游投诉统一受理机制，着手建立“1+n”全域旅游联合执法机制，依法严厉打击非法经营、价格欺诈和强迫消费等行为，营造诚实守信的旅游消费环境。

第四，抓好脱贫攻坚和农村农业工作。全面贯彻中央一号文件精神，推进农业供给侧结构性改革，增加绿色优质农产品供给，落实各项精准脱贫和强农惠农政策，促进农业增效、农民增收、农村增绿。

突出抓好精准脱贫。实施农村脱贫攻坚大会战，把产业扶贫放在第一位，积极探索产业脱贫的路子，组织贫困户参加农民专业合作社，因地制宜发展特色产业，增加贫困户收入。统筹安排并管好用好帮扶资金，加快推进贫困村基础设施建设，发展村集体经济，落实到村到户、精准有效的帮扶措施，努力实现“九有”，即村村有特色产品、有加工厂、有种养基地、有小市场、有电商、有农家乐、有升级版合作社、有小花园、有村庄规划。今年确保47个贫困村、4941户21076人达到脱贫标准。

大力发展农业生产。重点发展现代化、规范化、标准化的种植业和养殖业，加强科技创新引领，提升农产品质量和农产品安全水平，提高农业综合效益。扎实推进高标准基本农田建设，稳定粮食种植面积和粮食生产能力。培育新型农业经营主体和服务主体，推进现代特色农业示范区和“双高”糖料蔗基地建设，做大做强优势特色产业，促进一产二产三产融合发展，切实提高农业产业化水平。植树造林3万亩，稳定森林覆盖率。

扎实推进水利建设。推进洪潮江水闸、总江水闸和旺盛江水库除险加固工作，加快海湾新城乾江围、合浦县百曲围海堤标准化建设，开展南流江城区河势控制工程等河流治理项目建设。实施19个水库移民新村建设项目，保障按时足额发放水库移民后扶直补资金。

加快发展县域经济。深入实施大县城战略，加快合浦县城和铁山港中心城区建设，推进合浦县新型城镇化示范县建设。支持合浦工业园区扩园发展，促进合浦工业扩大规模。进一步完善铁山东港产业园道路、码头等基础设施，加快推进已签约项目尽快落地、形成新产能。统筹推进县区基础设施建设，完善产业布局，推动县区提升传统产业、发展特色产业，增强县域经济实力。

第五，积极发展现代服务业。优化服务业结构，提升服务业的效益和质量，补齐产业发展短板。

加快发展商贸等服务业。尽快出台商贸行业扶持发展政策，设立市级商贸流通业发展扶持专项资金，促进商贸业提升发展。制定批零住餐行业企业奖励办法，加强商贸企业入统工作，挖掘批发零售住宿餐饮行业发展潜力。推动软件和信息技术服务等新兴服务业加快发展。进一步优化金融环境，继续抓好“引金入北”工作，深化政银企合作，充分发挥金融服务经济社会发展的作用。

加快实施电商北海工程。以北海高新区为依托，发展2家电子商务产业园，培育5家以上自治区级电商示范单位，开拓农村电商、旅游电商、跨境电商等领域。促进互联网+批发、互联网+传统百货等商业模式加快发展。利用第三方电商平台，搭建线上特产馆、线下服务中心。积极推进跨境电商体验及跨境商品保税直销中心项目建设。加强电商扶贫工作，加快合浦县与阿里巴巴开展农村淘宝项目合作，推进合浦县创建电子商务进农村综合示范县工作。

加快化解房地产库存。房地产业既要防风险、去库存，又要惠民生、作贡献。要加强房地产市场监管和整顿，规范开发、销售、中介等行为。采取积极有效措施，调整优化房地产供给结构，落实相关政策，继续推动房地产去库存，促进房地产市场平稳健康发展。积极推动房地产与休闲旅游、健康养生养老等产业融合发展。

第六，做大做强海洋经济。发挥海洋资源优势，拓展蓝色经济空间，加快发展海洋产业，奋力舞起海洋经济发展的龙头，为再造一个“海上北海”打牢基础。

加快发展海洋产业。开展海洋经济普查，编制《北海市海洋产业发展规划》。加快发展临海工业、海洋运输、船舶修造等产业，增强海洋产业综合实力。提升发展传统渔业，推进海洋渔船更新改造项目和南沙船队建设，鼓励发展远海和远洋渔业。发展优势特色水产养殖，推广生态健康养殖模式，提高水产养殖效益。加快恢复发展南珠产业，重振南珠品牌。积极发展海洋信息、港航服务、海洋科技服务等海洋服务业。拓展海洋产业发展领域，探索发展海洋文化体验、海洋健康体育、邮轮经济等新兴业态。

加快建设北海海洋产业科技园区。设立海洋产业发展基金，重点支持海洋产业园区基础设施建设，支持海洋科技研发和成果转化、海洋经济创新平台建设。促进已签约的研究机构及企业加快进驻并实际运营，加快建设国家海洋局第四海洋研究所。抓紧完成“中民远洋北海项目”落地的前期工作，积极开展与台湾的渔业合作。加快推进“国家科技兴海示范基地”、营盘中心渔港、中国-东盟现代渔港经济区建设。

第七，加强交通基础设施建设。坚持优质发展、适度超前原则，更加重视交通基础设施建设和公共交通发展，加快完善立体交通网络，拓展对外开放通道，提升城市发展的承载能力。

加强公路设施建设。确保贵合高速公路北海段建成通车，加快建设松旺至铁山港东岸高速公路、广西滨海公路山口支线，开工建设G209合浦绕城公路、公馆至常乐公路，开展广西滨海公路大风江至高德段和关井至营盘段、铁山港滨海大道工程前期工作，筹划改造北铁一级公路、S210乌家至西场公路。

加强铁路设施建设。加快建设合湛铁路，启动建设玉铁铁路铁山港客运站，积极推进铁山港至石头埠铁路支线、铁山港至啄罗铁路支线前期工作，筹划建设合浦至铁山港铁路、沙河至铁山港东岸铁路支线和北海火车北站综合交通枢纽。开工建设北海动车所，争取北海至南宁区间的动车按照设计时速达速运营，加密北海火车站每天开行的动车车次。

加强港口设施建设。加快建设铁山港榄根作业区泊位、神华国华广投北海能源基地石头埠作业区泊位和铁山港航道三期工程。开工建设铁山港公共执法码头，开展铁山港北暮作业区港池、和润项目配套码头、石化专用液体化工码头等项目前期工作。

第八，大力推进改革创新。把创新摆在发展全局的核心位置，不折不扣落实中央、自治区的改革部署，推进一批人民群众关注以及能够解决制约发展问题的改革措施，增强发展的新动力。

推进重点领域改革。以推进供给侧结构性改革为主线，落实“三去一降一补”任务。深化简政放权、放管结合、优化服务改革。深化商事制度改革，全面推行“六证合一、一照一码”登记制度，加快电子营业执照和全程电子化登记管理改革。调整完善市辖区财政体制。深化国税、地税征管体制改革，加强“营改增”后续管理。深化国资国企改革，推动平台企业加快转型和重组，加强和规范国有企业监管，完善支持、引导非公有制经济加快发展的政策环境。深化医药卫生体制改革，全面启动城市公立医院综合改革。深化机关事业单位养老保险制度改革。深化城市管理综合执法体制改革，实现住房城乡建设领域行政处罚权相对集中行使。深化公安改革，加强能力建设，创新体制机制。深化农村综合配套改革，全面推进农村土地承包经营权确权登记颁证和农村土地“三权分置”工作，完成国有林场改革任务。加快推进供销合作社综合改革，增强发展活力和为农服务能力。积极推进其他领域的改革。

增强创新发展能力。尽快出台《北海市实施创新驱动发展的意见》及配套文件，加快完善创新机制。加强创新平台建设，推动北海高新区打造创新型特色园区，加快建设自主创新示范区；推广创客空间、创业社区等新型孵化模式，扶持建设一批众创空间，搭建创业孵化服务平台。设立企业创新引导基金，落实鼓励创新发展的税收优惠政策，支持一批创新型企业加快发展，着力打造技术优势产业。推广政府与社会资本合作模式（PPP），支持企业通过资本市场拓宽投融资渠道。

推动科技创新。发挥科技创新在全面创新中的引领作用，加大对科技创新的投入，建立科技成果转化引导激励机制，促进科技成果转化，推动科技和经济紧密结合。完善资助和奖励政策，提高专利成果的质量和数量。推动科技型企业运用资本市场，开展专利质押融资，促进金融与科技融合。培养和引进一批创新创业人才，推动科技创新、人才集聚与产业升级联动发展。

第九，不断扩大开放合作。落实自治区“四维支撑、四沿联动”部署，加强区域交流合作，促进加工贸易倍增和创新发展，切实发挥北海在北部湾经济区扩大开放中的龙头作用，加快开放发展步伐。

提升开放合作平台。进一步加强产业园区基础设施建设，创新体制机制，促进园区形成新的集聚效应和增长动力。推动北海出口加工区进一步完善综合保税仓库，更新封关围网、监控、卡口等海关监管设备设施，加强与海关、国检等相关部门的沟通协调，争取尽快升格为综合保税区；争取纳入国家第二批企业增值税一般纳税人资格试点，加快创建国家级高新技术产品入境再制造/全球维修示范区，力争年内正式挂牌。推动北海工业园区升级为国家级经济技术开发区、铁山港（临海）工业区升级为自治区级园区，支持北海高新区扩区扩容。加快建设铁山东港产业园，促进湛江-北海粤桂北部湾经济合作区上升到两省区的层面推进，打造开放合作新平台。

实施加工贸易倍增计划。用足用好CEPA先行先试政策，率先在全区开展与港澳合作先行先试，加快发展服务贸易；规划建设澳门-北海葡语系国家产业园，吸引葡语系国家的投资者到北海开展贸易及产品精深加工；以北海出口加工区为核心区域，创建国家进口贸易促进创新示范区。加大对高新技术产品的政策支持力度，推动加工贸易骨干企业实现倍增。促进石化产品进一步扩大贸易规模和后续精深加工。建设中西部进出口水（海）产品加工交易和展示中心。

增强口岸开放能力。加强与国家部委的沟通对接，尽快实现铁山港扩大开放的正式验收。在全区率先实施国际贸易“单一窗口”模式，切实提高通关便利化水平。加强与现代贸易龙头企业合作，建立国际贸易综合服务平台，积极申报水果、冰冻水产品、可屠宰活牛、种苗及可再生资源的指定口岸，加快构建综合口岸体系。

拓宽多维发展空间。落实国务院最近批复的《北部湾城市群发展规划》，积极参与北部湾城市群建设，主动加强与各兄弟城市的全面合作，共建共保洁净海湾，共同打造面向东盟的开放高地。加强与东盟国家的交流合作，积极对接发达国家先进生产力，扩大与港澳台的沟通对接，深化与泛珠三角的区域合作，拓展发展空间、增强发展活力，推动北海开放发展。

第十，掀起招商引资热潮。充分发挥全市重大项目策划与推进领导小组和六个推进工作组的统筹协调作用，切实做好新常态下招商引资工作，实现招商引资重大突破，力争新签项目合同投资额550亿元以上、招商引资实际到位资金415亿元以上。

开展精准有效招商。强化项目策划，完善信息平台，加强宣传推介，提高招商引资实效。突出产业招商，围绕发展壮大主导产业、优势产业和实施“四大会战”，着力抓好产业园区项目布局和招商引资工作。着眼于完善基础设施，拓展和延伸产业链，壮大产业集群，谋划储备一批好项目、大项目。积极面向珠三角和港澳台，面向央企、民企开展招商。拓宽招商视域，只要对北海的发展有利、符合法律法规、符合国家产业政策的项目，都可以引进。既要努力争取上级对招商引资和项目建设的支持，也要着力推进获支持的项目落地建设，切实改变重争取轻推进、重招商轻落实的做法。注重引进外资，支持外资企业发展壮大，促进实体经济发展。

创新招商引资方式。推行专业招商、委托招商、产业链招商、企业招商等模式，开展多种形式的招商引资。组建专业招商团队，开展精准招商、重点招商。加强与各地商会的沟通联系，发挥商会的作用，拓展招商渠道。在市权范围内制定招商引资优惠政策，完善招商引资的协同机制和激励机制，落实考核办法和奖励措施，发挥县区、产业园区和职能部门的招商引资作用和积极性，实行全员招商、全天候招商、走出去招商。坚持储备项目抓前期、在谈项目抓签约、签约项目抓开工、开工项目抓进度、投产项目抓达产，确保项目引得来、落得下、出成效。

优化招商引资服务。坚持招商与留商并重，建设法治化的市场营商环境。强化服务理念，简化审批程序，优化投资环境，积极回应企业经营者的意见建议，及时帮助企业解决遇到的困难，大力支持企业扩大发展。继续以“四定”办法推进项目建设，进一步完善领导班子成员联系推进重点项目制度。严格落实问责制度，对不负责任、推诿扯皮和损害投资者合法利益、影响项目引进的问题，要严肃查处。

第十一，努力营造自然生态。始终坚持绿色发展，严格落实环境保护“党政同责、一岗双责”，加强环境保护、治理和生态修复，营造山清水秀的自然生态。

加强环境突出问题整治。深刻汲取合浦部分沿海乡镇采石场破坏生态环境问题的教训，举一反三，针对中央环保督察组反馈的督察意见，以及群众反映强烈的问题，疏堵结合、标本兼治，全面加强治理，特别是要对合浦采石场进行彻底整治并加快实施生态修复。持续落实“水十条”“大气十条”“土十条”，建立健全生态环境保护长效机制，保持良好的环境质量，切实提高城市环境的宜居性。

加强水资源保护。落实《广西壮族自治区饮用水水源保护条例》，加强牛尾岭水库、洪潮江水库污染整治和水质修复，开展市区备用（第二）水源输水工程前期工作，强化地下水资源保护，推进冯家江和南流江流域生态治理，着力保障群众饮水安全。

加强蓝色海湾整治。综合整治地角内港、外沙内港、电建和南氵万渔港环境，集中整治非法围填海和圈占海滩涂、非法采砂等破坏环境问题，严厉打击电捕鱼、高压水枪捕捞沙虫等非法捕捞行为。加强山口红树林保护区、合浦儒艮自然保护区、涠洲岛珊瑚礁生态系统的保护和修复。制定并落实“海洋防污”应急预案，加强对廉州湾和入海江河、陆源入海排污口、海水浴场、港口和近海海域的监测和综合整治。

第十二，繁荣发展社会事业。繁荣发展先进文化，不断提升市民素质和社会文明程度，营造团结和谐的社会生态。

加快发展先进文化。加快图书馆新馆、工人文化宫改扩建等项目建设，推进村级公共服务中心建设。开展“文艺惠民周周演”“文艺精品月月演”，丰富群众文化生活。鼓励文艺创作，打造北海特色艺术精品。完善文化产业发展专项资金管理办法，出台民间博物馆等文化产业扶持政策，培育骨干文化企业，加强文化院团建设，积极推动《碧海丝路》商演。加强文物的保护利用，提高非物质文化遗产保护传承水平，扎实推进海上丝绸之路申遗工作。加强新闻出版、广播电视工作，提高主流媒体的影响力和传播力。加强文化市场监管，促进文化市场繁荣有序。

加快发展教育事业。推动义务教育均衡发展，加大力度发展农村教育，力争合浦县、海城区、银海区通过自治区义务教育均衡发展督导评估。加快普及高中阶段教育，着力提升教育质量。出台扶持高等院校发展政策，支持桂电北海校区、北海艺术设计学院扩建，妥善处理北航北海学院办学问题，完善现代职业教育基础能力建设。发展多元普惠性幼儿园，统筹做好特殊教育和农村留守儿童、进城务工人员随迁子女教育工作。支持民办教育，鼓励社会力量兴办教育。实施教育领域高层次人才引进培养计划，加快建设教育人才公寓。

加快建设健康北海。继续做好国家级精神卫生综合管理试点工作，全面启动国家级结核病分级诊疗和综合防治服务模式试点。完成市儿童医院、市妇幼保健院保健中心项目主体工程，启动市中医医院新院区项目前期工作。实施基层医疗卫生服务体系行动计划，加强社区医疗机构建设，推进基本公共卫生计生服务均等化。落实全面两孩政策,促进人口均衡发展。

加快发展体育事业。贯彻《全民健身计划纲要》，广泛开展群众体育活动，做到月月有赛事。承办环广西自行车公路赛始发站比赛、2017（北海）国际青少年足球邀请赛和国际城市围棋联赛总决赛。建设20个村级篮球场，安装25条健身路径，力争市体育馆完成主体工程。推动体育冬训基地建设，做大做强足球培训产业。积极发展沙滩海洋体育。

第十三，着力提升社会保障水平。统筹做好高校毕业生、城镇就业困难人员、被征地农民和农村转移劳动力就业创业工作。实施全民参保登记计划，健全城镇职工、城乡居民养老保险和医疗保险制度，实现养老保险、医疗保险全覆盖。建设跨省异地就医即时结算平台，开展复合式医保付费方式改革，提高医保基金使用效益。完善城乡居民最低生活保障机制和社会救助制度，及时调整和执行低保标准，保持全区的中上水平。加强保障性住房建设，加快城中村和农村危旧房改造，棚户区住房改造开工4971套（户），基本建成公共租赁住房3188套、棚户区住房改造600套。保障妇女儿童合法权益，发展残疾人和老龄事业。

第十四，为民办好十件实事。在完成自治区下达的为民办实事工程项目的基础上，切实办好十件群众所急、百姓所需的实事，项目总投资26.2亿元,年内完成投资7亿元。

一是建成市人民医院银滩分院门诊楼并投入使用，完成市第二人民医院搬迁工程主体建设，开工建设涠洲岛旅游区医院。增加医疗服务设施，方便市民看病就医，缓解看病难问题。

二是完成北京八中北海分校新校区主体工程。引进北京八中办学，进一步满足群众对优质教育资源的需求。

三是建成市花公园和体育休闲公园并开园迎客。利用市区铁路沿线的防护用地，规划建设“北海绿廊”，其中今年建成市花公园和体育休闲公园。

四是实施中山公园和长青公园升级改造。优化公园布局，完善和增设休闲、游乐设施，亮化、彩化、美化公园环境。

五是开工建设海丝路广场。项目建设用地约5.7公顷，构建铁路以南商业文化中心，为市民提供新的休闲、娱乐及商业服务场所。

六是开工建设市博物馆和市档案馆。建筑面积4.1万平方米,进一步完善城市功能，促进文化事业发展。

七是开工建设青少年宫和妇幼活动中心。建筑面积4万平方米，增加青少年和妇女儿童综合活动场所，促进青少年、妇女、儿童事业的健康发展。

八是完成北海大道（南珠大道至迎宾大道、迎宾大道至合浦工业大道段）升级改造。按照市政主干道Ｉ级标准改造升级，全长11.7公里，促进主城区与合浦县城一体化发展。

九是开工建设西南大道东延线。按照城市主干道Ｉ级标准建设，总长5.2公里，构建进出主城区的新通道。

十是大力推进宜居乡村惠民工程。建设10个农村电子商务服务站点，建成300公里自然村硬化道路，完成50个自然村改厨、改厕工作，举办文化惠民演出180场次。

第十五，全面推进依法治市。落实各项法治建设工作任务，加快建设法治北海。扎实开展“七五”法治宣传教育，强化市民法治意识。完善法律援助制度，为群众提供更加便捷、优质的法律服务。

加强和创新社会治理，加快推进平安北海（三期）建设，实施北海公安“六大工程”，持续不断打击传销，进一步提升群众安全感满意度，维护社会和谐稳定。切实做好信访维稳工作，妥善处理信访突出问题，加强仲裁调解，积极化解各种矛盾纠纷。

落实安全生产工作各项举措，深化重点行业领域安全专项整治，有效遏制重特大安全生产事故发生。全面创建广西食品安全城市，加强食品药品安全监管。加强应急能力建设，完善工作机制，抓好防灾减灾救灾工作。大力支持国防和军队建设，做好优抚安置和双拥工作。

四、进一步加强政府自身建设

政府工作贵在担当、重在实干。完成今年各项目标任务，必须进一步加强政府自身建设，尽心尽力做好工作，努力建设人民满意的法治政府和服务型政府。

认真落实“两个责任”。按照全面从严治党的要求，落实好从严治党各项任务，把全面从严治党要求体现在政府工作全过程。牢固树立政治意识、大局意识、核心意识、看齐意识，同以习近平同志为核心的党中央保持高度一致，严守党的政治纪律和政治规矩，严肃党内政治生活，加强党内监督，不折不扣贯彻落实党中央、自治区党委和市委的决策部署。

严格依法履行职责。落实《北海市法治政府建设具体实施方案》，坚持法定职责必须为、法无授权不可为，执行政府部门权力清单和责任清单制度，严格按照法定权限和程序行使政府权力。配合市人大开展立法工作，加快推进《北海市涠洲岛生态环境保护条例》以及城市综合管理等领域的立法。贯彻执行市人大及其常委会的决议决定，接受人大及其常委会的法律监督、工作监督，支持人民政协履行政治协商、民主监督、参政议政职能，做好人大代表建议和政协提案办理工作。

努力提高行政效能。加大简政放权力度，切实提高行政效能和服务水平。落实行政审批“三项制度”，加强监督投诉管理，简化行政审批流程，提高审批效率。完善政府公共服务平台，推进“互联网+政务服务”，实现政务服务事项网上办理。整合乡镇政务服务资源，打通服务群众的“最后一公里”。

不断改进机关作风。加强机关作风建设，严明工作纪律，增强服务意识，提高工作效率。大力弘扬敢于负责、勇于担当精神，着力解决少数干部不作为、乱作为、不善为等问题。宽容干部在工作中特别是改革创新中的失误，为实干者鼓劲、为改革者撑腰、为担当者担当。加强对重点工作、重点项目和“四大会战”组织实施的督促检查，加大行政问责力度，确保各项工作落到实处。

始终坚持廉洁从政。认真履行“一岗双责”，加强党风廉政教育，贯彻执行各项党内法规。强化行政监察、审计的监督作用，加强对公共资金、公共资源、国有资产使用的监管。实践运用“四种形态”，坚持把纪律和规矩挺在前面，持之以恒落实中央八项规定和自治区纪委“十个严禁”，坚决反对“四风”，加强扶贫领域监督执纪问责，深入推进党风廉政建设和反腐败工作，营造风清气正、干事创业的良好政治生态。

各位代表！

春天是播种的季节，播种理想，播种希望，更要播种力量。回顾过去，北海发展已经很好；检视现状，北海还能更好；展望未来，北海应该最好。让我们紧密团结在以习近平同志为核心的党中央周围，在自治区党委、政府和市委的正确领导下，在市人大、市政协的支持和监督下，不用扬鞭自奋蹄，撸起袖子加油干，在建设创新型开放城市的征程中，创造无愧于历史的业绩，交出让人民满意的答卷！
